# Incremental Forming of Natural Fiber-Reinforced Polypropylene Composites: Considerations on Formability Limits and Energy Consumption

**DOI:** 10.3390/ma18122688

**Published:** 2025-06-07

**Authors:** Antonio Formisano, Dario De Fazio, Giuseppe Irace, Massimo Durante

**Affiliations:** Department of Chemical, Materials and Production Engineering, University of Naples Federico II, P.le V. Tecchio 80, 80125 Napoli, Italy; dario.defazio@unina.it (D.D.F.); giuseppe.irace@unina.it (G.I.); mdurante@unina.it (M.D.)

**Keywords:** incremental forming, polypropylene, flax, hemp, formability, forces, energy

## Abstract

Incremental sheet forming originated as an excellent alternative to conventional forming techniques for incrementally deforming flat metal sheets into complex three-dimensional profiles. Recently, its use has been extended to polymers and composites. Among these, the use of natural fiber-reinforced composites is increasing considerably compared to synthetic fiber-reinforced composites, due to the availability and unique properties of natural fibers in polymer applications. One of the dominant thermoplastics used as a matrix is polypropylene. This experimental study focuses on the incremental forming of natural fiber-reinforced polypropylene composites. Cones and spherical caps were manufactured from composite laminates of polypropylene reinforced with hemp and flax long-fiber fabrics. The formability limits, observed through failures and defects, as well as the forming forces, power, and energy consumption, were investigated to examine the feasibility of incremental forming applied to these composite materials; based on the results obtained, it is possible to say that the process can manufacture components with not very high wall angles but under low load conditions and allowing to limit the energy impact.

## 1. Introduction

Incremental sheet forming (ISF) fits into the context of highly flexible technologies, such as additive manufacturing, stimulated by recent significant advances in computer applications in manufacturing [1]. This materials processing technology exhibits several unique characteristics, such as reduced tooling, cycle time, and cost.

The principal concept of ISF in its basic variant involves the progressive deformation of a clamped material sheet by a simple, non-dedicated forming tool, guided by a CNC machine, which follows a path to incrementally deform the sheet into the final shape [2]. Thanks to the layered manufacturing principle typical of rapid prototyping, it allows for the high customization of small-batch, non-axisymmetric sheet products [3], with potential applications in aerospace, automotive, and other fields [4,5].

Early research on ISF has mainly focused on metals and their alloys, with several articles and reviews published on the topic. For example, initial overviews of the process [6] have been followed by more recent literature reviews detailing scientific progress and future developments [7,8,9]. Other papers have focused on specific topics such as formability [10], deformation [11], failure mechanisms [12], and analyses of forming forces [13]. Additionally, other works have investigated the applications of ISF for metal parts [14,15,16,17,18].

Recently, researchers have shown increased interest in hard-to-form non-metallic materials, particularly thermoplastic polymers [19,20,21]. Thermoplastics exhibit desirable properties (light weight, strength, corrosion resistance, and cost-effectiveness, among others), making them widely used for mass production [22]. ISF can represent a viable alternative to conventional processes for these materials, which require repetitive heating, shaping, and cooling actions [23,24]. However, progress in the ISF of composites has not been significantly reviewed or documented. Some preliminary studies have been conducted on the incremental forming of sandwich panels [25] and composite materials [26], as well as on advances in ISF of common polymer-based composite materials reinforced with glass [27] and carbon fibers [28]. Nonetheless, it is evident that there is an urgent need to deepen knowledge in this area, especially as industries seek alternative, sustainable, and cost-effective solutions for processing composite materials [29].

An area of recent significant interest, both in research and on an industrial scale, is the manufacturing of polymer composites using natural fibers as reinforcement [30]. These fibers represent an inexpensive, biodegradable, renewable, and nontoxic alternative to the most common synthetic fillers (glass and carbon fibers) [31]. They enhance certain properties of commercial polymers, reduce energy consumption, and make them semi-biodegradable [32]. Hemp and flax are the strongest and stiffest natural fibers, as well as two of the most popular and widely available fibers in European countries. They are composed of several elementary fibers that are glued together by a middle lamella mainly composed of pectin [33]. These fibers exhibit low density and high specific stiffness compared to glass or aramid fibers and are commonly used for the manufacture of biocomposites [34]. Flax is widely cultivated in countries with cold and moist climates that promote its short growing cycle, such as Canada, Russia, France, and Belgium. Hemp has a very rapid growth cycle of only 3.5 months, high dry biomass production (4–5 times higher than that produced by a forest of the same area in one year), and high carbon storage potential [35].

Polypropylene (PP), thanks to its high chemical and wear resistance, excellent mechanical properties, ease of processing, and cost-effectiveness [36], is the world’s second-most widely produced synthetic polymer. It is employed in various industrial applications, including automotive parts, reusable containers, packaging, and laboratory equipment [37]. PP is strongly considered for advanced composites in aerospace, civil, and automotive fields [38] and currently dominates as a matrix for natural composites, along with polyethylene and polyvinyl chloride [39].

This work presents an experimental campaign of ISF tests for the manufacture of cones and spherical caps, starting from hemp and flax fiber-reinforced PP laminate composites. By observing failures and defects, it determines the formability of these composite materials. Additionally, energy considerations are made through the evaluation of forming forces. In doing so, the work contributes to enriching knowledge about the feasibility of ISF of natural fiber-reinforced composites, identifies potential applications in industrial fields and outlines directions for future research, with particular emphasis on hot topics such as sustainable manufacturing and circular economy.

## 2. Materials and Methods

**Figure 1:**
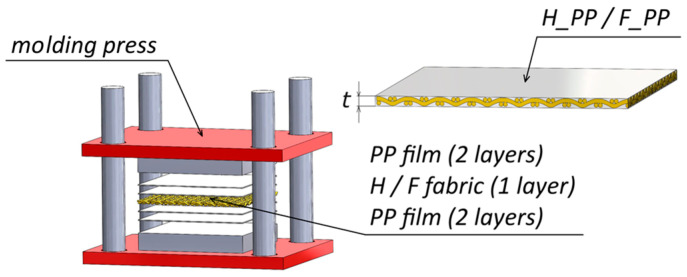
Schematic of the molding process.

The laminates employed in this study were natural long hemp and flax fiber-reinforced PP composites, labeled as H_PP and F_PP, respectively, with a thickness *t* = 2.2 mm. They were manufactured through a molding process at 200 °C for a total time of 5 min, using PP films (supplied by GDC S.r.l. (Arzignano, Italy); thickness of 0.5 mm and density of 0.92 g/cm^3^) and hemp and flax fabrics, supplied by FIDIA S.r.l. (Milano, Italy)—Technical Global Services. The choice of the materials and of the process parameters described above were proved to achieve notable improvements in tensile and bending properties of flax and hemp fiber-reinforced PP composites compared to unreinforced PP laminates, even with a different stacking sequence [40]. The main properties of the fibers (both single unimpregnated yarn and fabric) are summarized in Table 1 and Table 2, while a schematic of the molding process and the layup is shown in Figure 1.

**Figure 2:**
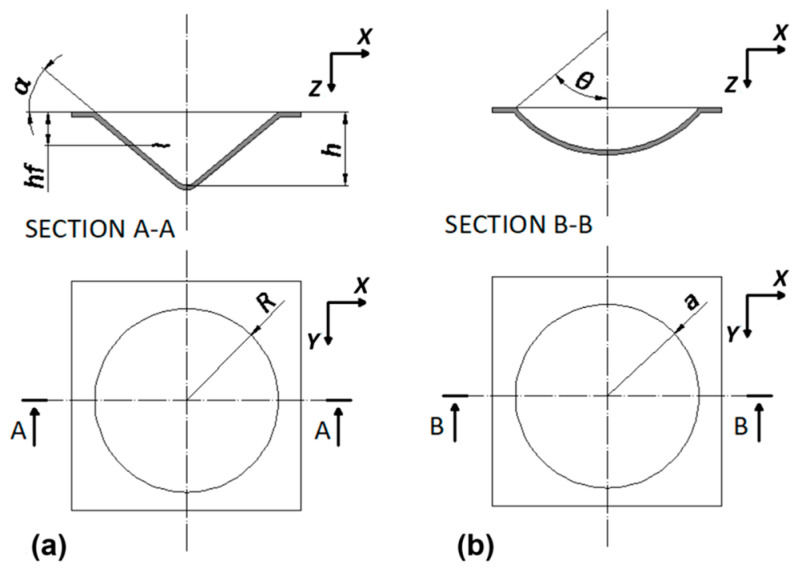
CAD representation of the components manufactured by SPIF process: (**a**) cones and (**b**) spherical caps.

Cones and spherical caps (two repetitions for each case) were manufactured using the simplest variant of the process, known as single-point incremental forming (SPIF). A CAD representation of the components is shown in Figure 2. In Figure 2a, *R*, *h* and *α* represent the base radius, height, and wall angle of the cones, respectively, while *hf* is the height at the potential point of failure. Additionally, *a* and *θ* in Figure 2b denote the base radius and polar angle of the spherical caps.

**Figure 3:**
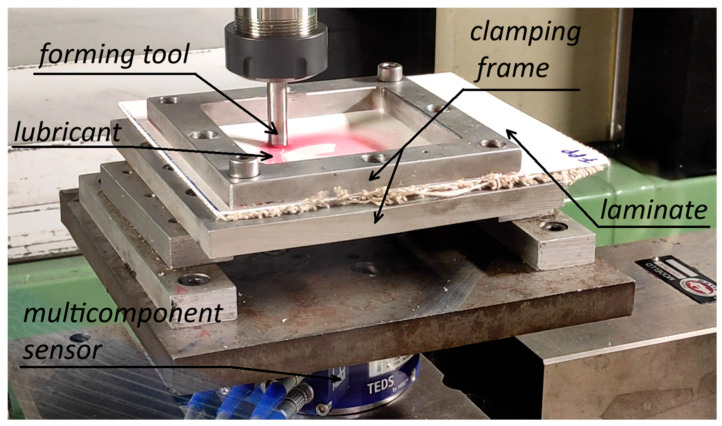
Execution of an FF_P cone SPIF test.

**Figure 4:**
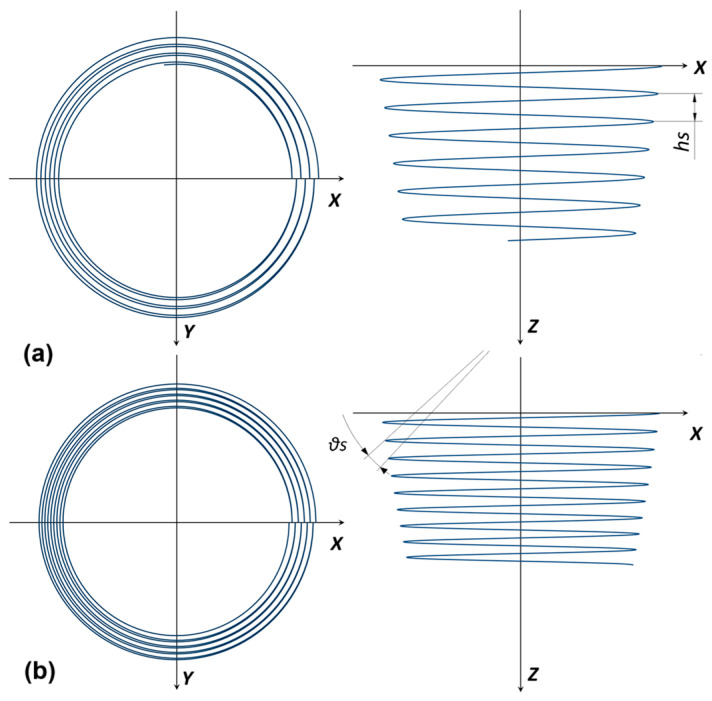
Toolpaths for the SPIF process: (**a**) cones and (**b**) spherical caps.

The forming tests (see an example for the manufacture of an F_PP cone in Figure 3) were conducted using a C.B. Ferrari high-speed four-axis vertical machining center (C.B. Ferrari S.r.l., Mornago, Italy). This machine drove the forming tool, a non-rotating stainless-steel stylus with a hemispherical head 10 mm in diameter, to progressively deform the composite laminates. The laminates were secured using a clamping frame with a square working area of 100 × 100 mm^2^. The forming tool followed alternating helical toolpaths (the turns alternated in anticlockwise and clockwise directions; see their not-to-scale representation in Figure 4), with constant vertical and angular steps down (*hs* and *θs*, i.e., the vertical and angular distance covered after one complete turn of the toolpaths, respectively) and at a nominal speed of *v* = 1000 mm/min.

Despite recent innovations in the ISF of biocomposites that provided for localized heating [41], the SPIF process was conducted at room temperature to preserve its flexibility and ease of use. To reduce the probability of encountering failures and defects, the tests were carried out under lubricated conditions using the Boelube 70104 (100A) synthetic lubricant, developed by Boeing and supplied by Orelube (Bellport, NY, USA).

The experimental campaign aimed to evaluate various types of failures and defects during the SPIF tests to collect information on the formability limits of SPIF applied to these composite laminates. Additionally, it provided information on the forming forces required for the manufacturing process. Three forces (*F_X_*, *F_Y_*, and *F_Z_*; the modules of the force acting in the sheet plane, *F_XY_*, and of the total forming force, *F_TOT_*, which is the resultant of the forces acting between the forming tool and the laminate, were obtained as a combination of the three components) and one moment (*M_Z_*) were acquired at a frequency of 50 Hz using the K-MCS10 multicomponent sensor (HBK World, Southfield, MI, USA) (see Figure 3), equipped with the QuantumX MX840B data acquisition system and the Catman Easy AP software V5.6.1. These forces were also used to evaluate the process power profile and energy consumption. This choice of using the forces, instead of evaluating the energy requirement, is justified by the fact that the actual energy required for the process is only a small proportion of the total energy consumed, as most of the energy requirements are due to additional functions of the equipment [42].

## 3. Results and Discussion

This section reports the results and discussion of the experimental campaign, subdivided into two parts. The first part describes the feasibility of the SPIF process and the formability limits reached for the two laminate types, while the second part discusses the forming forces, power, and energy required for the process. Due to the very good repeatability of the results, for the sake of conciseness and brevity, only the representative curves and average values of the features investigated are reported.

### 3.1. Feasibility of the SPIF Process and Formability Limits

The first part of the experimental campaign involved the manufacture of cones, represented in Figure 2a, with *R* = 40 mm and three different *α* values (30°, 40°, and 50°); for the toolpath (see Figure 4a), *hs* = 1 mm. All the components showed no twisting, due to the alternating nature of the toolpath. This significantly reduces the probability of twisting because the twist produced after a turn is almost completely recovered in the next [43], as observed in the ISF of metal [6] and polycarbonate components [44]. Additionally, the lack of instability and wrinkling indicates non-severe working conditions [45].

**Figure 5:**
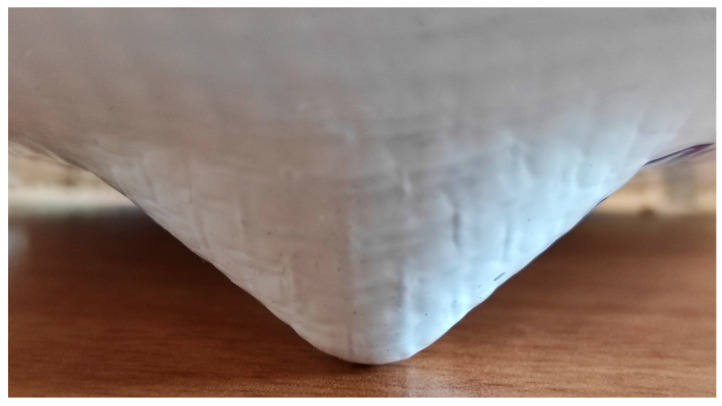
H_PP cone (*α* = 40°).

Regarding formability limits, the cones were sound and had good surface quality for both fibers at *α* = 30° and *α* = 40°, reaching their maximum allowable heights *h* (approximately 22 mm and 32 mm, respectively). Figure 5 shows an H_PP cone at *α* = 40°.

**Figure 6:**
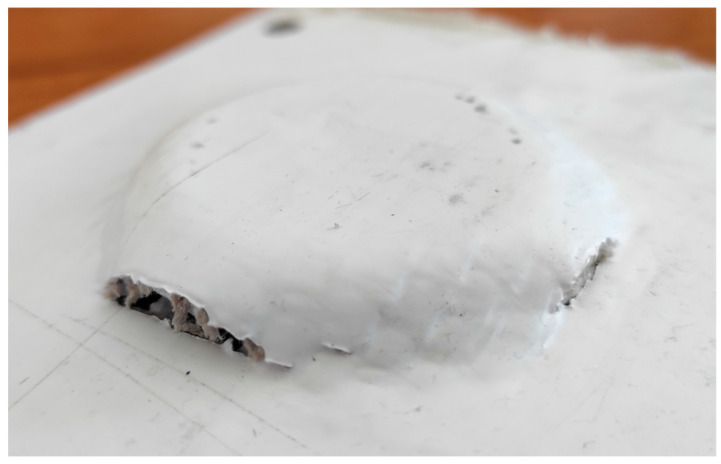
H_PP cone (*α* = 50°).

**Figure 7:**
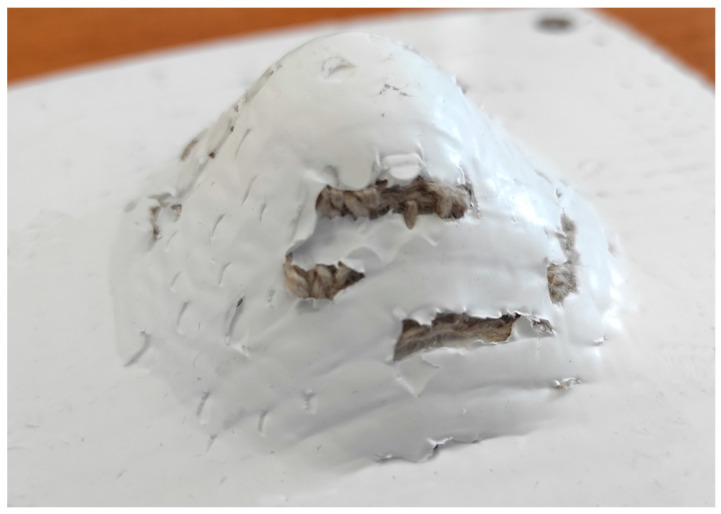
F_PP cone (*α* = 50°).

Failures occurred at *α* = 50° for both fiber types. For H_PPs (see Figure 6), the failure was perpendicular to the fabric and occurred at *hf* ≈ 20 mm. For F_PPs (see Figure 7), the failure occurred at higher heights (*hf* > 30 mm) and primarily affected the matrix. These differences could be due to the mechanical behavior of the laminates, influenced by the efficiency of the fiber–matrix adhesion, in turn depending on the molding process.

Despite the process not allowing for very high wall angles, it can be used for applications such as shaping stiffening ribs for panels in the automotive, aviation, and naval fields [46]. In this context, spherical caps were manufactured (see Figure 2b). They had an *a* = 40 mm and two different *θ* values (40° and 50°); for the toolpath, *θs* = 1° (see Figure 4b).

**Figure 8:**
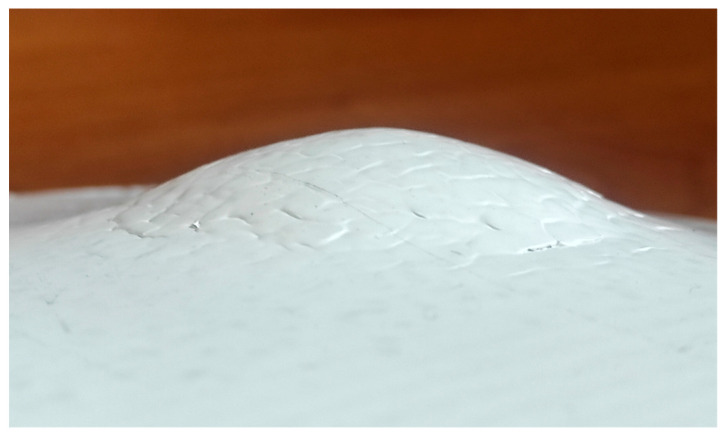
F_PP cap (*θ* = 50°).

The caps were sound and had good surface quality for both fibers and at both *θ* values. Figure 8 shows an F_PP cap with *θ* = 50°.

### 3.2. Considerations on Forces, Power, and Energy

**Figure 9:**
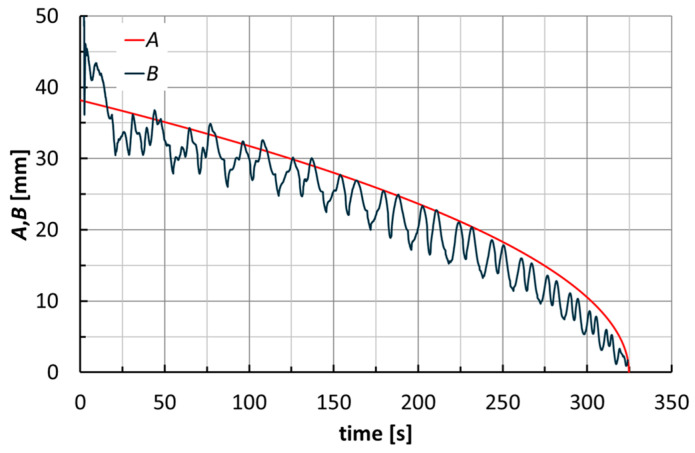
Toolpath (*A* curve) and absolute value of the ratio between *M_Z_* and *F_XY_* (*B* curve) for an F_PP cone (*α* = 40°).

A first observation can be made by analyzing the curves in Figure 9 (related to an F_PP cone at *α* = 40°), where *A* represents the current radius of the spiral toolpath and *B* represents the absolute value of the ratio between *M_Z_* and *F_XY_*. Excluding the first turns of the toolpath (which can be considered a transition phase of the process), curve *A* can be seen as the envelope of curve *B*. This confirms the incremental nature of the ISF process because the *B* ratio represents the lever arm of *F_XY_* that generates *M_Z_*.

**Figure 10:**
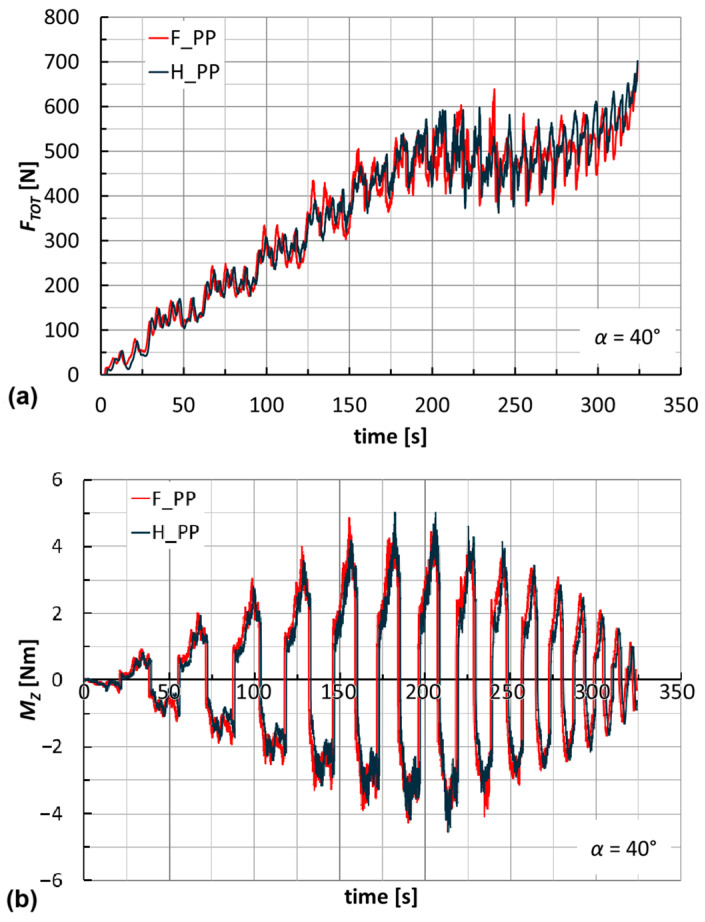
Trends of (**a**) *F_TOT_* and (**b**) *M_Z_* by varying the laminate (*α* = 40°).

**Figure 11:**
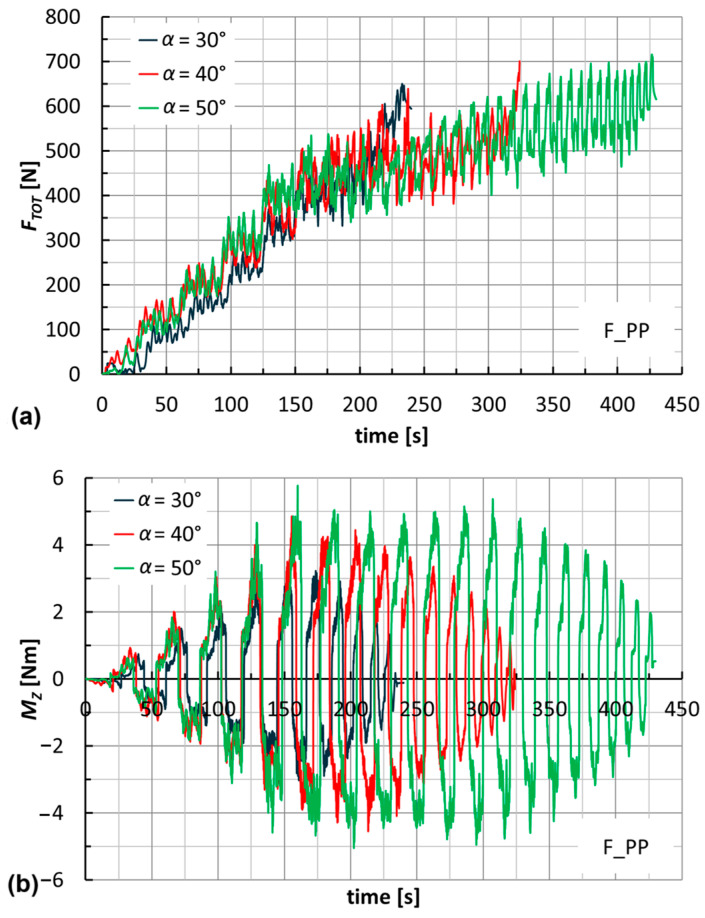
Trends of (**a**) *F_TOT_* and (**b**) *M_Z_* by varying the wall angle (F_PP).

The trends of forces and moments reflect the alternating nature of the toolpaths (this also justifies the fluctuations of *B* in Figure 9). They are very similar for the two laminates; see, for example, Figure 10 for the manufacture of cones at *α* = 40°. Their maximum values slightly increase with *α* (see Figure 11 for F_PP laminates), and as anticipated earlier, translate into non-severe working conditions that suggest low energy impact for this manufacturing process. The maximum values of *F_TOT_* and of the module of *M_Z_* (see Table 3 and Table 4 for H_PP and F_PP tests, respectively), both reached for F_PP at *α* = 50°, were equal to 716 N and 5.8 Nm, respectively. Table 3 highlights an anomaly concerning the maximum value of *F_TOT_*, justified by the premature failure for H_PP at *α* = 50°.

The total power, *P_TOT_*, was obtained by separately considering the contributions of *F_XY_* and *F_Z_* (*P_XY_* and *P_Z_*, respectively), according to the following equations:

*P_TOT_* = *P_XY_* + *P_Z_*, (1)

*P_XY_* = *F_XY_* × *v*, (2)

*P_Z_* = *F_Z_* × *v_Z,m_*, (3)

where *v_Z,m_* is the mean value of the speed along the *Z* axis, calculated as the ratio between the total vertical displacement and the time taken to describe it.

For the process under examination, the speeds in the *XY* plane and along the *Z* axis (*v_XY_* and *v_Z_*, respectively) are not constant because they depend on the actual slope of the spiral toolpath. However, two simplifications were applied in Equations (2) and (3), to use two constant values of speed. Specifically, *v* was used in place of *v_XY_* in Equation (2) because the low value of *hs* compared to the spiral radii translates into a low slope (a large discrepancy between *v* and *v_XY_* occurs only when the last turn of the toolpath reaches the vertex of the cone). Similarly, it is possible to replace *v_Z_* with *v_Z,m_* in Equation (3), especially considering that these are very low speeds and may not significantly contribute to the total power and energy.

**Figure 12:**
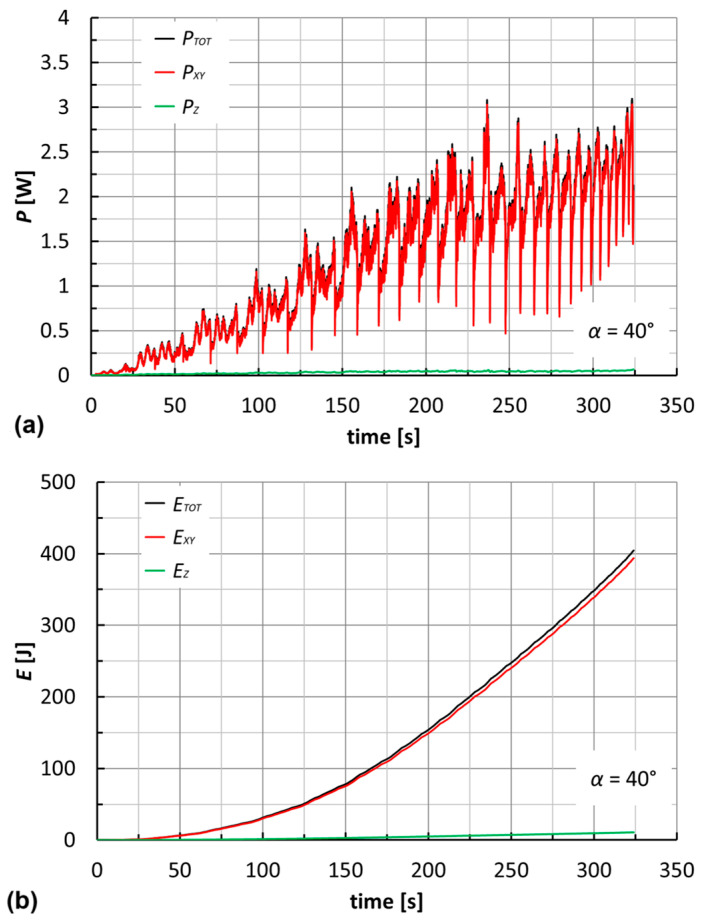
Trends of (**a**) power and (**b**) energy for an F_PP laminate (*α* = 40°).

Figure 12a reports the power curves for an F_PP laminate at *α* = 40°. It is evident that the contribution of *P_Z_* is nearly irrelevant, in line with the above considerations in Equation (3). Therefore, it is possible to make a good estimate of the total power through an assigned constant process parameter (*v*) and the monitoring of *F_XY_*. Alternatively, and according to the suggestions from Figure 9, it would be necessary to know the angular speed law (not constant) and to monitor *M_Z_*. Quantitatively, the powers reached are very low for all cases (under 4 W; see Table 3 and Table 4), due to the low *F_XY_* and *v* values.

The energies (*E_TOT_*, *E_XY_*, and *E_Z_*; see Figure 12b for an F_PP laminate at *α* = 40°) were determined as the time integral of the power curves. The Riemann integral was used, with a regular partition of the time equal to the period of acquisition of the forces (0.02 s).

Just as with the powers, *E_TOT_* is almost equal to *E_XY_*. Moreover, the maximum values for the two geometries are reached for F_PP laminates at *α* = *θ* = 50° (about 610 and 440 J, respectively; see Table 4).

## 4. Conclusions

This experimental research outlines the feasibility and energy implications of the single-point incremental forming process applied to laminates of flax and hemp fiber-reinforced polypropylene composites.

The first part of the work focuses on the manufacture of cones with different wall angles (30°, 40°, and 50°) and demonstrates that the process, carried out at room temperature and without dedicated dies, ensures sound cones up to 40° while failures occur for 50° cones; such formability limits allow for the manufacture of stiffening ribs like spherical caps.

The analysis of forces and moments highlights that the process requires low load levels (under the severest working conditions, less than 720 N and 6 Nm). Additionally, the force-based evaluation of powers and energies reveals the very low energy impact of the process under examination, which is of great interest from a sustainable manufacturing perspective.

Future research could extend the investigation of the process parameters that influence the incremental formability of natural fiber-reinforced composites, including carrying out more specific varying wall angle tests, as well as compare this process with other forming processes in terms of energy efficiency. Additionally, it could include the extended pre- and post-forming mechanical characterization of the panels, numerical simulations of the process, the investigation of the geometrical accuracy of the components, and the feasibility of remolding panels after incremental forming to promote circularity.

## Figures and Tables

**Table 1:** Properties of hemp fibers.

Single Unimpregnated Yarn	
Tensile strength [MPa]	507
Tensile modulus [GPa]	18.40
Elongation at break [%]	3.27
Density [g/cm^3^]	1.40
Fabric	
Tex [g/km]	334
Mass per unit area [g/m^2^]	380

**Table 2:** Properties of flax fibers.

Single Unimpregnated Yarn	
Tensile strength [MPa]	512
Tensile modulus [GPa]	21.40
Elongation at break [%]	3.27
Density [g/cm^3^]	1.50
Fabric	
Tex [g/km]	320
Mass per unit area [g/m^2^]	320

**Table 3:** Maximum values of the features investigated from H_PP tests.

Case	Cones			Spherical Caps	
	*α* = 30°	*α* = 40°	*α* = 50°	*θ* = 40°	*θ* = 50°
*F_TOT_* [N]	638	686	560	389	464
Module of *M_Z_* [Nm]	3.7	4.8	5.8	2.5	3.8
*P_TOT_* [W]	2.3	2.8	2.7	1.2	1.8
*E_TOT_* [J]	191	362	327	232	436

**Table 4:** Maximum values of the features investigated from F_PP tests.

Case	Cones			Spherical Caps	
	*α* = 30°	*α* = 40°	*α* = 50°	*θ* = 40°	*θ* = 50°
*F_TOT_* [N]	650	701	716	434	473
Module of *M_Z_* [N]	3.3	4.9	5.8	3.1	4.1
*P_TOT_* [W]	2.4	3.1	3.3	1.4	1.9
*E_TOT_* [J]	187	404	608	271	441

## Data Availability

The original contributions presented in the study are included in the article, further inquiries can be directed to the corresponding author.

## Abbreviations

The following abbreviations are used in this manuscript:

ISF	Incremental sheet forming
CNC	Computerized numerical control
PP	Polypropylene
H_PP	Hemp fiber-reinforced polypropylene composites
F_PP	Flax fiber-reinforced polypropylene composites
*t*	Laminate thickness
SPIF	Single-point incremental forming
CAD	Computer-aided design
*R*	Base radius of the cones
*h*	Height of the cones
*α*	Wall angle of the cones
*hf*	Height at the potential point of failure of the cones
*a*	Base radius of the spherical caps
*θ*	Polar angle of the spherical caps
*hs*	Vertical distance covered after one complete turn of the toolpaths
*θs*	Angular distance covered after one complete turn of the toolpaths
*v*	Nominal toolpath speed
*F_X_*	Module of the forming force along the *X* axis
*F_Y_*	Module of the forming force along the *Y* axis
*F_Z_*	Module of the forming force along the *Z* axis
*F_XY_*	Module of the forming force acting in the *XY* plane
*F_TOT_*	Module of the total forming force
*M_Z_*	Module of the moment around the *Z* axis
*A*	Current radius of the spiral toolpath
*B*	Absolute value of the ratio between *M_Z_* and *F_XY_*
*P_TOT_*	Total power
*P_XY_*	Power associated with *F_XY_*
*P_Z_*	Power associated with *F_Z_*
*v_Z,m_*	Mean value of the speed along the *Z* axis
*v_XY_*	Speed in the *XY* plane
*v_Z_*	Speed along the *Z* axis
*E_TOT_*	Total energy
*E_XY_*	Energy associated with *F_XY_*
*E_Z_*	Energy associated with *F_Z_*
